# Calcaneo‐stop for paediatric idiopathic flexible flatfoot: High functional results and return to sport in 644 feet at mid‐term follow‐up

**DOI:** 10.1002/jeo2.70182

**Published:** 2025-02-20

**Authors:** Simone Silva, Tullia Tavernini, Alessandro Bruschi, Luca Andriolo, Giulia Guizzardi, Margherita Serra, Gino Rocca, Giuseppe Filardo

**Affiliations:** ^1^ Clinica Ortopedica e Traumatologica 2, IRCCS Istituto Ortopedico Rizzoli Bologna Italy; ^2^ SC Ortopedia e Traumatologia Pediatrica, IRCCS Istituto Ortopedico Rizzoli Bologna Italy; ^3^ Department of Medical and Surgical Sciences University of Bologna Bologna Italy; ^4^ Faculty of Biomedical Sciences Università della Svizzera Italiana Lugano Switzerland

**Keywords:** arthroereisis, calcaneo‐stop, idiopathic flexible flatfoot, sport

## Abstract

**Purpose:**

Idiopathic flexible flatfoot (IFF) is a frequent condition in children. Patients refractory to conservative treatments may benefit from surgical procedures. The aim of this study was to evaluate clinical outcomes and sport activity levels in a large cohort of paediatric patients treated with calcaneo‐stop (CS) for the symptomatic IFF.

**Methods:**

A single‐centre retrospective study was conducted using an institutional database that prospectively collected clinical outcomes of patients treated with CS for symptomatic IFF. The procedure included the implantation of a cancellous screw through the talus, which was subsequently removed after 2 years. A total of 644 feet (336 consecutive patients) followed up to a mean of 41.3 ± 6.7 months after implant removal were included. Foot pain and sport activity were assessed.

**Results:**

A successful outcome, defined as the presence of a painless, corrected foot together with patient satisfaction, was obtained in 94% of the patients, while 35 feet were considered failed. Extracurricular sport participation was possible in most patients after CS screw implantation (55%) and fully recovered after implant removal (77%). No activity level increase was shown compared to baseline. The only factor that correlated significantly with the incidence of failures was the occurrence of adverse events (*p* = 0.001), which negatively influenced also the sport activity level (*p* = 0.008). Females obtained lower Tegner scores compared to males (3.5 vs. 5.0, *p* < 0.0005).

**Conclusions:**

CS procedure provided highly satisfactory clinical results at mid‐term follow‐up. Full sport activity level was recovered after screw removal and a 94% favourable outcome was obtained in terms of foot pain relief as well as patient and parents satisfaction.

**Level of Evidence:**

Level IV, case series.

## Graphical abstract

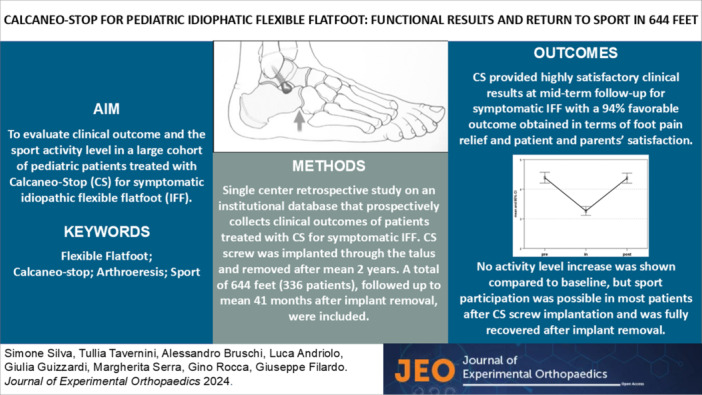

**Abbreviations**
ANOVA: analysis of variance
BMI: body mass index
CS: calcaneo‐stop
IFF: idiopathic flexible flatfoot

## INTRODUCTION

Idiopathic flexible flatfoot (IFF) is a condition characterized by loss of the medial plantar arch height, pronation of the forefoot, and hindfoot valgus, which affects 4% of 10 years old children, with half of them presenting painful symptoms [17]. Despite the common occurrence of IFF, its clinical impact is controversial, and it remains to be proven if the biomechanical alterations connected with IFF could compromise the activity level of these young patients [2, 6]. There are still no univocal indications for treatment, and no data suggest that the treatment of IFF in the child will prevent the development of symptomatic flatfoot in the adult, even with the more invasive surgical options [23, 28]. Due to these controversies, the leading indication for treatment is the presence of painful symptoms [12]. Conservative treatments consist of kinesiotherapy and orthotics, useful in relieving pain but without evidence of biomechanical correction. As the plantar arch develops until age 10, conservative treatments are attempted in younger children, while surgical treatment is usually considered after this age to directly correct the biomechanical deformity [5, 9].

Different surgical procedures have been proposed over time to treat symptomatic IFF, with arthroereisis and osteotomies being the most used [16]. There are no clinical or radiological features leading to the surgical choice, and no study demonstrated the superiority of one procedure over the other [25, 30]. While osteotomies aim to correct the alignment or the lengths of foot bone segments, arthroereisis is a procedure aimed at limiting the pronation of the subtalar joint by using a screw (re‐adsorbable or non‐re‐adsorbable) [7, 30]. Two different kinds of arthroereisis have been suggested: endosinutarsal and esosinutarsal [29, 30]. The former, also called endorthesis, limits the pronation at subtalar level positioning the device inside the sinus tarsi, while the latter, also known as calcaneo‐stop (CS), consists of a screw inserted outside the sinus tarsi, in the calcaneus or in the tarsus [15, 21]. CS is the most common arthroereisis procedure currently performed, and several studies analysed its results, demonstrating a good clinical improvement [10]. Nevertheless, little is known about the influence of CS on sport activity.

The aim of this study was to evaluate the clinical outcome of CS with the hypothesis that it would lead to the recovery of function and sport activity level in a large cohort of paediatric patients affected by symptomatic IFF.

## METHODS

### Study design

This is a single‐centre retrospective study conducted using an institutional database that prospectively collects clinical outcomes of patients treated with the CS for symptomatic IFF. The study was approved by the Hospital Ethics Committee and Internal Review Board of the Rizzoli Orthopaedic Institute, Bologna, Italy (prot. no. 10963), and informed consent was obtained from all patients.

### Surgical procedure and rehabilitation protocol

The indication for surgical treatment with CS was symptomatic unilateral or bilateral IFF with foot pain or abnormal premature muscle fatigue during normal gait or during sport activities. All surgical procedures (both related to IFF and to comorbidities) were performed by experienced surgeons, specialized in paediatric orthopaedics, from the same paediatric orthopaedic division of a referral orthopaedic centre.

**Figure 1:**
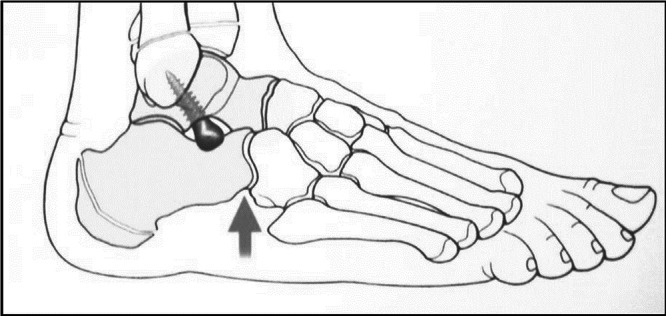
Technical representation of the CS inserted in the talus preventing pronation and reestablishing plantar arch, as the grey arrow indicates. CS, calcaneo‐stop.

After general or spinal anaesthesia, patients were placed in a supine position and a thigh tourniquet was applied. A 2 cm skin incision was performed in correspondence with the sinus tarsi, and after a blunt soft tissue dissection, having care not to damage the peroneus brevis tendon and to protect the sural nerve, an entry hole on the anterior aspect of the lateral process of the talus was done using a sharp trocar under fluoroscopic control. A 6.5 mm titanium alloy cancellous screw (Spherus®, Gruppo Bioimpianti) was then inserted under fluoroscopic control through the talus at a 35° direction in the sagittal plane and 45° in the coronal plane, while maintaining the foot in the corrected position. The screw length (ranging from 20 to 40 mm) was determined according to the patient's talus dimensions. The device rationale relies on the spherical head of the screw impinging with the calcaneus, thus resulting in a mechanical correction of the foot, as well as a proprioceptive trigger [27]. Moreover, the optimal correction was chosen by slightly tightening or loosening the screw, checking the residual pronation clinically by stressing the head of the V metatarsus simulating weight‐bearing (Figure 1). Finally, haemostasis was done after tourniquet release, then the skin was sutured, and compressive medication was applied. Patients were usually discharged from the hospital the day after surgery.

Post‐operatively, patients were left non‐weight‐bearing for 3 days, then gait was allowed using orthopaedic non‐articulated walking boots and crutches for a total of 25 days. Return to sport was gradually permitted after boots removal, starting from low‐impact sports, like swimming or cycling, to a complete return to the favourite sport within three months.

Implant removal was performed after a minimum of 2 years or after a two‐size growth of the foot, and it was done in the same surgical setting. Afterwards, patients could tolerate weight‐bearing with the support of crutches from the day after surgery for a total of 10 days and return to sport was fully permitted within maximum 30 days after surgery.

### Patient selection and evaluation

Patients included for the current analysis were those consecutively treated for symptomatic IFF with CS by the equipe of the Paediatric Orthopedic Division (Rizzoli Orthopaedic Institute, Bologna) and with at least 2 years of follow‐up after screw removal. Patients with a rigid flatfoot from bone coalitions or with a flatfoot as a result of a congenital anomaly or syndrome were excluded, as well as patients with neuromuscular disorders, post‐traumatic deformities, severe spine disorders, or patients with malignancy or juvenile arthritis.

All patients were evaluated preoperatively and at a follow‐up of at least 24 months from implant removal (41.3 ± 6.7 months). Baseline data such as age, sex, body mass index (BMI) and related orthopaedic comorbidities were recorded for each patient at the time of CS screw implantation. Any surgery of the foot combined with CS (i.e., percutaneous Achilles tendon lengthening, tibialis anterior tenodesis, tibialis posterior tendon retention or accessory tarsal scaphoid removal) was recorded, as well as any combined surgery not involving the foot. At the final follow‐up, each patient was evaluated through a telephone questionnaire in order to assess the presence of foot pain during daily or sport activities, as well as the overall level of satisfaction regarding the treatment. The following questions were asked to patients: ‘Do you feel pain in the treated foot during daily or sport activities? Are you satisfied with the results achieved after the treatment?’. In addition, the type and level of sport activity before the implantation, during the treatment (after implantation and before screw removal), and after CS screw removal were evaluated with the Tegner activity scale. A further distinction was also made between school sports and extracurricular sports. Adverse events and failures were also documented. The treatment was deemed to have failed if the patient complained of persistent foot pain or was dissatisfied with the treatment from the patients or their parents at the time of follow‐up evaluation or if the patients required further surgery for the IFF.

### Statistical analysis

All continuous data were expressed in terms of the mean and the standard deviation of the mean, and the categorical data were expressed as frequency and percentages. The Shapiro–Wilk test was performed to test the normality of continuous variables. The Levene test was used to assess the homoscedasticity of the data. The repeated measures analysis of variance (ANOVA) with the Sidak test for multiple comparisons was performed to assess the differences at different follow‐up times of normally distributed variables. The Friedman test, followed by the Wilcoxon test pairwise comparisons with Bonferroni correction, was performed to assess the differences at different follow‐up times of non‐normally distributed variables. The one ANOVA test was performed to assess the between‐group differences of continuous, normally distributed and homoscedastic data; the Mann–Whitney non‐parametric test was used otherwise. The ANOVA test, followed by the post hoc Sidak test for pairwise comparisons, was performed to assess the among‐group differences of continuous, normally distributed and homoscedastic data, the Kruskal–Wallis non‐parametric test, followed by the post hoc Mann–Whitney test with Bonferroni correction for multiple comparisons, was used otherwise. The Spearman rank Correlation was used to assess correlations between numerical scores and continuous data. The Pearson chi‐square was evaluated using an exact test that was performed to investigate relationships between grouping variables. The Logistic regression was performed as multivariate analysis to determine which factor independently influenced the outcome. For all tests, *p* < 0.05 was considered significant.

All statistical analysis was performed using SPSS v.19.0 (IBM Corp.).

## RESULTS

**Figure 2:**
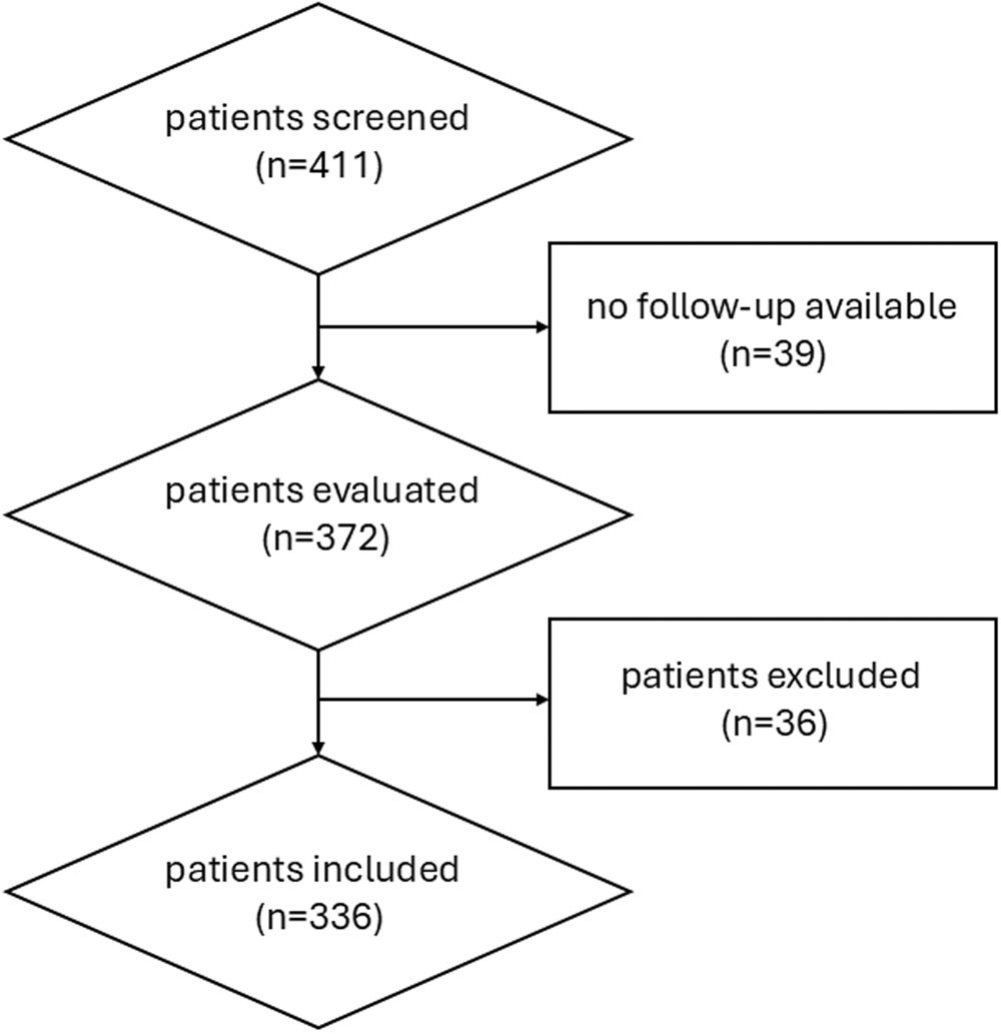
Patients inclusion flowchart.

Of the initial 411 patients considered, 336 consecutive patients, 308 affected by bilateral symptomatic IFF and 28 affected by monolateral symptomatic IFF, were included in the current analysis according to inclusion/exclusion criteria (Figure 2), for a total of 644 feet treated between 2014 and 2018. A summary of clinical and demographic characteristics of data is reported in Table 1. The mean treatment duration between screw implantation and removal was 26.8 ± 7.5 months. The evaluation was performed at a mean follow‐up period from implant removal of 41.3 ± 6.7 months.

**Table 1:** Clinical and demographic information of patients.

Patient parameters	Data
Age at CS implantation, mean ± SD	11.7 ± 1.3
At CS removal, mean ± SD	14.0 ± 1.4
At follow‐up, mean ± SD	17.4 ± 1.3
Sex (*n*, %)	
Female	197 (58.6%)
Male	139 (41.4%)
BMI, mean ± SD	20.6 ± 3.5 Kg/m2
Basal sport level (*n*, %)	
Scholar	93 (27.7%)
Extracurricular	243 (72.3%)
Orthopaedic comorbidities (*n*)	61 (*p* = 0.145)
Scoliosis	33
Genu valgum	22
Patellar instability	2
Other	4
Feet involved (*n*, %)	644
Monolateral	28 (4.3%)
Bilateral	616 (95.7%)
Combined foot surgery (*n*, feet)	54 (*p* = 0.122)
Achilles tendon lengthening	48
Tibialis posterior tendon retention	4
Tibialis anterior tenodesis	2
Combined surgery (*n*)	21
Bilateral asymmetric physeal closure	19
Patellar realignment	2

Abbreviations: BMI, body mass index; CS, calcaneo‐stop; SD, standard deviation.

Overall, more than 94% of the treated patients obtained a successful outcome, which is defined as the presence of a painless, corrected foot, together with patient satisfaction. On the other hand, 20 patients were considered failures according to the definition, resulting in 35 failed feet. In detail, pain persisted in a total of 27 feet, whereas 4 patients with bilateral IFF expressed dissatisfaction regarding the treatment, reporting that they would not repeat the procedure.

No severe adverse events were reported, while mild adverse events, including complaints of post‐operative pain or personal discomfort during the time of treatment, were reported in 18 patients for a total of 31 feet.

After the implantation of the CS screw (during CS treatment), the sport activity level evaluated with the Tegner scale decreased significantly (*p* = 0.001) from a baseline level of 4.4 ± 2.4 to a level of 3.4 ± 1.9. Among the 243 patients practising extracurricular sports before treatment, 57 (23.4%) switched to only practising scholar sports during this time, while the other 186 (76.6%) resumed their regular sport activity after the mean of 3.4 ± 3.3 months after device implantation (Table 2).

**Table 2:** Number and rate of patients with different sport levels before CS, with CS implanted and after CS removal.

Sport level	Before CS	With CS	Post CS
Scholar only	93 (27.7%)	150 (44.6%)	77 (22.9%)
Extracurricular	243 (72.3%)	186 (55.4%)	259 (77.1%)

Abbreviation: CS, calcaneo‐stop.

**Figure 3:**
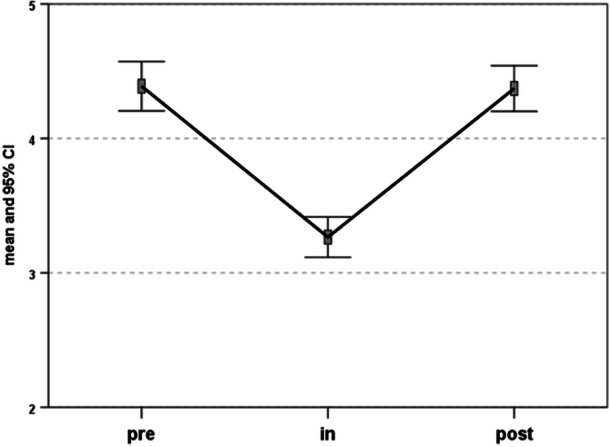
Tegner activity scale trend: Before implantation (pre); with the screw implanted (in) and after removal (post). CI, confidence interval.

After implant removal, the Tegner improved significantly with respect to the CS treatment time (*p* = 0.001) to a mean score of 4.4 ± 2.2 at the final follow‐up, reaching results comparable to the baseline level. The portion of patients also practising extracurricular sports increased after screw removal, reaching a total of 259 patients at the final follow‐up (Figure 3). A similar trend was observed when considering only the subset of patients who also participated in extracurricular sports. In this group, the Tegner score decreased from a baseline of 5.0 ± 2.2 to 4 ± 2.1 following device implantation. However, the score improved to a mean of 5.0 ± 2 after the device was removed.

Further analysis was performed to identify factors influencing the clinical outcome. The only factor which correlated significantly with the incidence of failures was the occurrence of adverse events (*p* = 0.001). Adverse events also negatively influenced the sport activity level (*p *= 0.008). Other factors were found to correlate with the sport outcome. Female patients obtained lower final Tegner compared to males (3.5 ± 1.4 vs. 5.0 ± 2.4, *p* < 0.0005). However, they both benefited from a similar improvement from baseline level to follow‐up. Moreover, compared to males, a significantly higher percentage of females did not practise any extracurricular sport before the implant and did not practice any sport besides scholar sports after treatment (16.0% vs. 4.7%). As well, females showed higher extracurricular sports discontinuation after implant removal (17.9% vs. 9.7%) compared to males (*p* < 0.0005). Age showed a tendency to correlate with the sport activity level, with younger patients presenting a higher improvement of the Tegner score, but without reaching statistical significance (*ρ* = −0.076, *p* = 0.055). The duration of CS treatment was correlated with the sport activity level, with a lower improvement in patients with longer time between implantation and removal (*ρ* = 0.078, *p* = 0.048). Finally, other factors like height, weight, BMI, and combined procedures did not influence the outcome after CS treatment.

**Figure 4:**
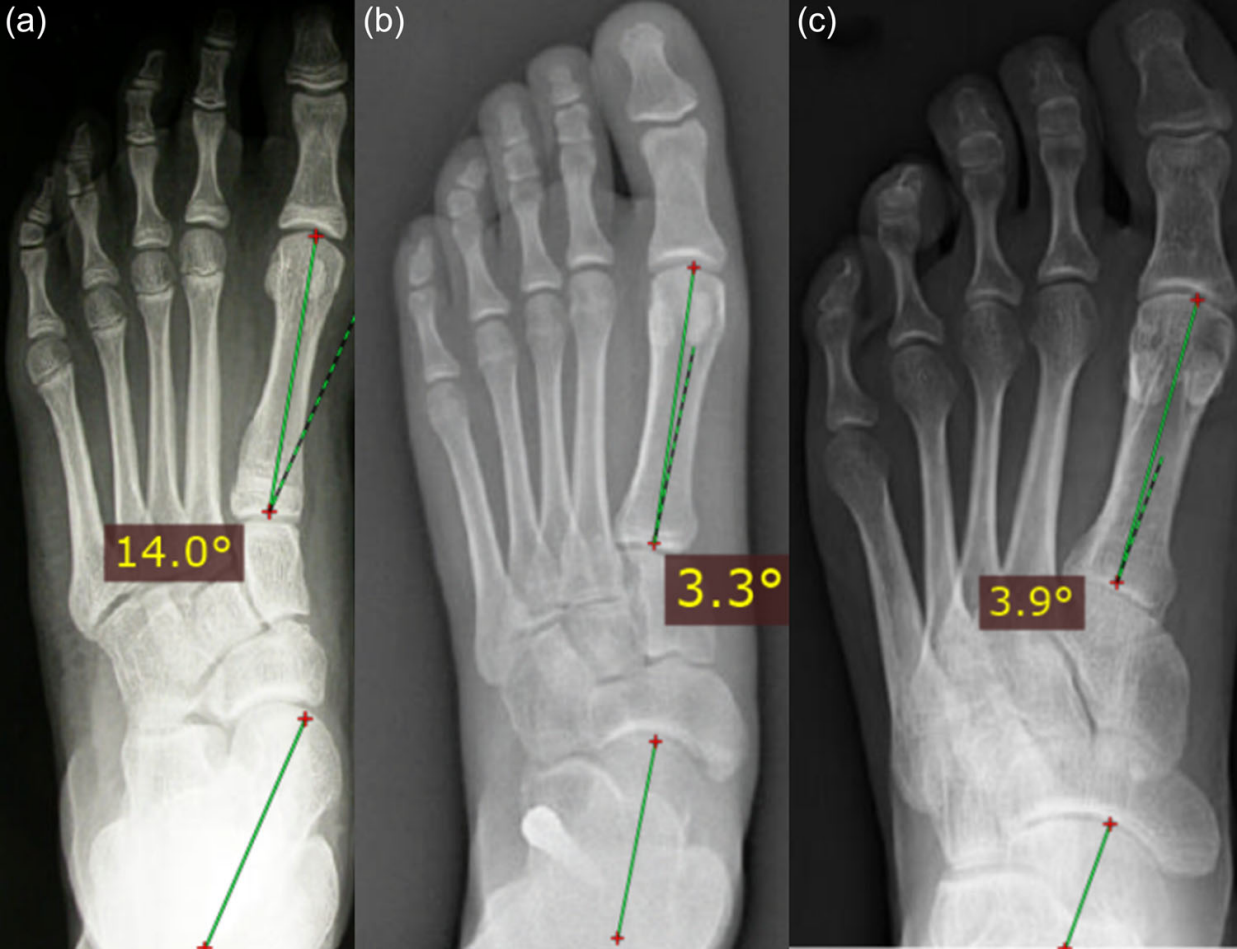
Talar–first metatarsal angle, between the lines drawn along the long axis of the talus and the first metatarsal in antero‐posterior radiographic view; before correction (a), with the screw implanted (b) and 11 years after screw removal (c).

**Figure 5:**
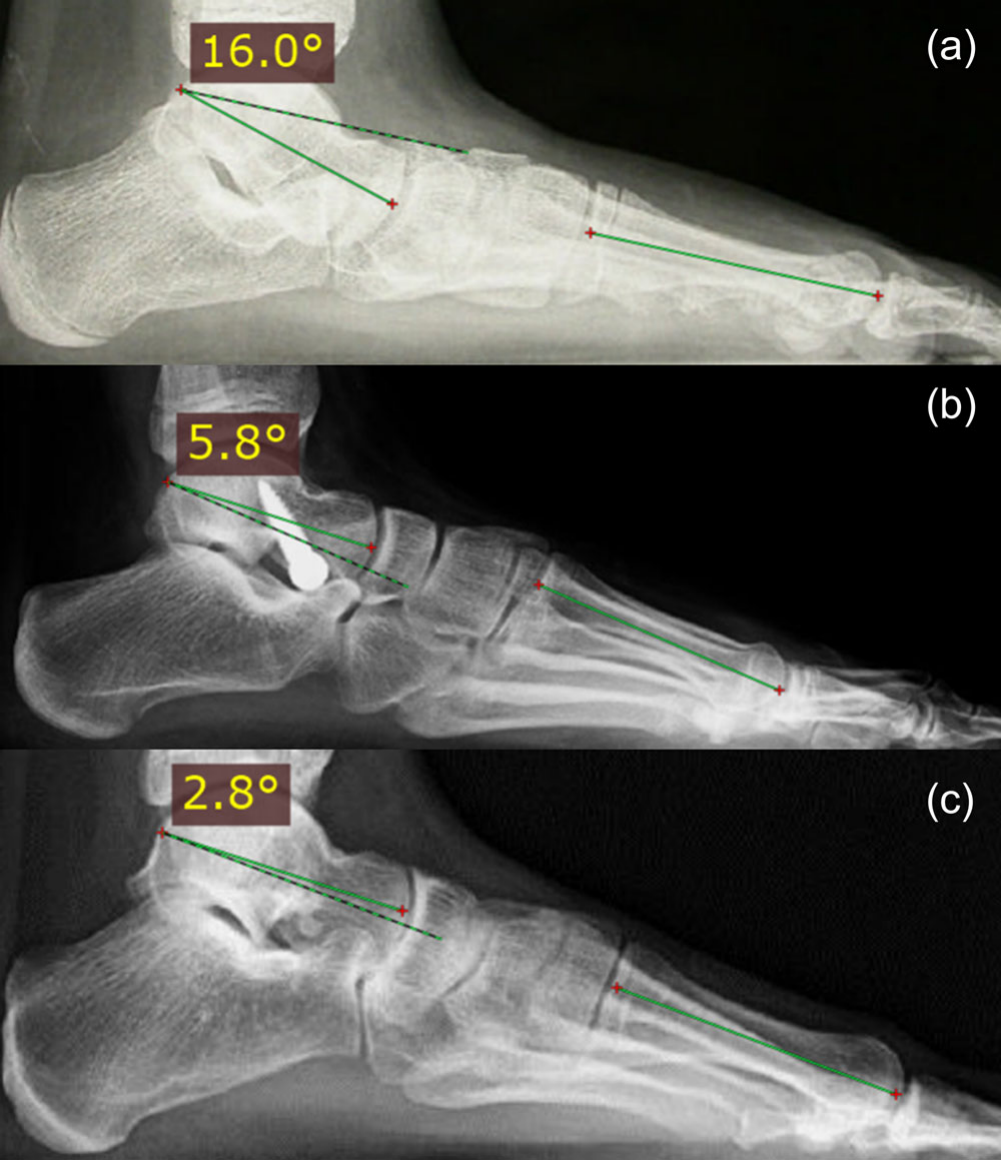
Talar–first metatarsal angle (Meary's angle), between the lines drawn along the long axis of the talus and the first metatarsal in lateral radiographic view, before correction (a), with the screw implanted (b) and 11 years after screw removal (c).

Although radiographs were not routinely utilized for determining treatment indications or monitoring patients, radiographic images have been included to illustrate the correction provided by the screw and how this correction was sustained after device removal (Figures 4 and 5).

## DISCUSSION

The main finding of this study is that subtalar arthroereisis with CS provided highly satisfactory clinical results at mid‐term follow‐up in paediatric patients suffering from symptomatic IFF. While no activity level increase was shown compared to baseline, sport participation was possible in most patients after CS screw implantation. It was fully recovered after implant removal confirming the hypothesis, and favourable outcome of 94% was obtained in terms of foot pain relief as well as patient and parents satisfaction.

Flatfoot represents a physiological condition during the first few years of life and, although IFF has a high prevalence in the young paediatric population, in most cases, it represents an asymptomatic condition. This may be a reason for parents concern but does not require intervention and is resolved spontaneously by adolescents [4, 5, 9]. However, a significant portion of paediatric patients with IFF complain of foot pain and altered foot functionality, which could result in a quality‐of‐life impairment with an altered gait and early fatigue of the foot during normal daily activities, especially sports‐related [13, 22]. The importance of sport activity in growing children has been well demonstrated in terms of health benefits, playing an important role in both children's mental and physical growth [8]. Nevertheless, only few studies investigated the relationship between paediatric IFF and sport [18, 19]. Moreover, many surgical procedures have been described for IFF, adding heterogeneity and increasing uncertainty when interpreting literature results on this important aspect in young patients' lives [1].

The standard CS technique with the screw implanted through the calcaneus, described in the 70s by Burutaran, was later modified by Castaman in 1985 with the screw implanted in the talus aiming to take advantage of an improved surgical procedure feasibility. Castaman's technique has been considered in the current study [3, 6]. The analysis of this large cohort of paediatric patients showed that CS is a safe, minimally invasive surgical procedure with few complications, able to provide satisfying clinical outcomes. Less than 6% of patients reported residual pain or dissatisfaction. This is in line with the results presented by De Pellegrin et al., which showed similar outcomes reporting an improvement in almost 94% of 398 patients treated with the CS screw inserted in the calcaneus [10]. Besides confirming the overall potential of the arthroereisis approach to improve symptoms and quality of life, the present analysis provides key results on the poorly investigated impact of the CS treatment in terms of patient activity level.

The analysis of sport activity of these patients revealed a decline in the sport level during the period when patients had the screw implanted. This could be explained by the fact that patients, even if allowed to practice their favourite sports, may take greater caution during the time they had the screw implanted. However, the sport level decrease during this time was transient and limited and most patients were able to perform some type of physical activities already 3 months after implantation, with only a small part of them complaining of implant‐related discomfort. Also, the second surgery to remove the screws allowed patients to swiftly return to physical activity. In this regard, this study showed that after the implant removal, the return to a sport activity reached a level equal to before the screw implantation. Despite the overall good outcomes reported, it should be underlined that the level of sport activity obtained was not superior to the pre‐treatment one, when patients sought treatment for symptomatic IFF. These results are in contrast with the study of Pavone et al., who analyzed the impact of standard CS procedure on sport in a selected cohort of 68 sportive patients affected by IFF and observed a substantial improvement in terms of mean sport activity time, passing from 2.5 h per week before surgery to 5.7 hours three years after surgery [26]. On the other hand, Martinelli et al., in a study on 49 patients affected by IFF and treated with subtalar arthroereisis showed good clinical results in terms of emotional status and footwear issues, but with no significant improvements regarding duration, frequency, and type of sporting activities [24]. The authors of this study suggested that the discrepancy between sport activity level and clinical results could be in part related to changes in life priorities and time demands throughout adolescence, resulting in a decline in sport activity participation. This is a phenomenon that we also witnessed regarding these patients when asked about their sport attitude. Moreover, this trend appears to be more evident among adolescent females, with females having a lower tendency towards extracurricular sport activities, especially after implant removal [11]. Overall, we observed that patients who used to practice sport activities before CS treatment returned to sport practice without pain after CS removal, whereas those who did not practice sport before did not start sport practice after CS removal, regardless of the clinical improvement obtained.

The main limitations of this study are the absence of a control group and the retrospective nature of the study design. The lack of radiological comparison pre‐ and post‐treatment represents another potential limitation, even though the clinical examination remains the standard method to evaluate CS treatment success. Moreover, it can be pointed out that the results of the current study are specific to the talus CS, and further studies should analyze and compare the results of the described procedure to the other arthroereisis approaches. Despite these limitations, this study provides important data on a large cohort of patients, all evaluated not only in terms of symptoms and satisfaction, but also investigating adverse events, failures, and the impact on sport both during and after treatment.

Overall, the main limitation of surgical procedures for addressing IFF is the lack of clear indications. Regrettably, it has been suggested that parents concerns may somewhat influence the management of this condition, hence contributing to its possible overtreatment [5, 14]. It should be therefore mandatory to support clinical decisions not only considering patients clinical examination but even more relying on the subjective symptoms reported by patients. Operative surgical interventions should be taken into consideration in IFF with relevant symptoms, after a period of observation and nonoperative management consisting of activity changes, stretching and eventually use of orthoses. However, these measures do not affect foot evolution [16, 20]. When considering surgical treatment of IFF, it is relevant to consider the severity of pain and how this affects daily activities, including participation in sports. Nevertheless, patients and parents must be informed that increased sport activity levels should not be expected. Thus, improvement of symptoms and deformity correction, rather than increased sport activity, should be the expected outcomes of the surgical treatment of IFF.

## CONCLUSIONS

Subtalar arthroereisis with CS provided highly satisfactory clinical results at mid‐term follow‐up in paediatric patients suffering from symptomatic IFF. No activity level increase was shown compared to baseline, but sport participation was possible in most patients after CS screw implantation and was fully recovered after implant removal. Finally, favourable outcome of 94% was obtained in terms of foot pain relief as well as patient and parents satisfaction.

## AUTHOR CONTRIBUTIONS

Conceptualization: Giuseppe Filardo. Methodology: Simone Silva, Tullia Tavernini, Alessandro Bruschi and Luca Andriolo. Data curation: Simone Silva, Alessandro Bruschi, Giulia Guizzardi and Margerita Serra. Writing—original draft preparation: Simone Silva and Alessandro Bruschi. Writing—review and editing: Luca Andriolo, Tullia Tavernini and Giuseppe Filardo. Supervision: Gino Rocca and Giuseppe Filardo. All authors have read and agreed to the published version of the manuscript.

## CONFLICT OF INTEREST STATEMENT

The authors declare no conflicts of interest.

## ETHICS STATEMENT

The study was approved by the Hospital Ethics Committee and Internal Review Board of the Rizzoli Orthopaedic Institute, Bologna, Italy (prot. no. 10963), and informed consent was obtained from all patients.

## Supporting information

Supporting information.

## Data Availability

The raw data supporting the findings of this study are available from the corresponding author upon reasonable request.
