# Supplementary material for: Calcaneo‐stop for paediatric idiopathic flexible flatfoot: High functional results and return to sport in 644 feet at mid‐term follow‐up
Source: J Exp Orthop. 2025 Feb 20;12(1):e70182. doi: 10.1002/jeo2.70182 (PMC11840700; doi:10.1002/jeo2.70182)
Supplement: Supplementary file 1 — Supporting information. [file JEO2-12-e70182-s001.doc]

STROBE Statement—Checklist of items Yes that should be included in reports of ***cohort studies***

|  | Item No | Recommendation |
| --- | --- | --- |
| **Title and abstract** | 1 | (*a*) Indicate the study’s design with a commonly used term in the title or the abstract  **Respected:** Yes  **Comment:** Stated in the abstract |
|  |  | (*b*) Provide in the abstract an informative and balanced summary of what was done and what was found  **Respected:** Yes  **Comment:** Stated in the introduction and methods section of the abstract |
| Introduction | | |
| Background/rationale | 2 | Explain the scientific background and rationale for the investigation being reported  **Respected:** Yes  **Comment:**  CS is the most common arthroeresis procedure currently performed, and several studies analysed its results demonstrating a good clinical improvement. Nevertheless, little is known about the influence of CS on sport activity. |
| Objectives | 3 | State specific objectives, including any prespecified hypotheses  **Respected:** Yes  **Comment:**  To evaluate clinical outcome and the sport activity level in a large cohort of paediatric patients treated with Calcaneo-Stop (CS) for symptomatic idiopathic flexible flatfoot (IFF). |
| Methods | | |
| Study design | 4 | Present key elements of study design early in the paper  **Respected:** Yes  **Comment:** Stated in the “Study Design” section |
| Setting | 5 | Describe the setting, locations, and relevant dates, including periods of recruitment, exposure, follow-up, and data collection  **Respected:** Yes  **Comment:** Stated in the “surgical procedure” section |
| Participants | 6 | (*a*) Give the eligibility criteria, and the sources and methods of selection of participants. Describe methods of follow-up  **Respected:** Yes  **Comment:** Described in the “Patient selection and evaluation” section |
|  |  | (*b*) For matched studies, give matching criteria and number of exposed and unexposed  **Respected:** No  **Comment**: Not a matched study |
| Variables | 7 | Clearly define all outcomes, exposures, predictors, potential confounders, and effect modifiers. Give diagnostic criteria, if applicable  **Respected:** Yes  **Comment:** Outcomes for every patient were comprehensively reported |
| Data sources/ measurement | 8* | For each variable of interest, give sources of data and details of methods of assessment (measurement). Describe comparability of assessment methods if there is more than one group  **Respected:** Yes  **Comment:** Described in “Patients selection and evaluation”. In particular variables of interest are reported and the source of data (clinical evaluation in outpatients clinics). No description of comparability is reported as there is just one group of patients. |
| Bias | 9 | Describe any efforts to address potential sources of bias  **Respected:** Yes  **Comment:** This has been indicated in the discussion section, reporting limitations. The main sources of bias of the study were the lack of a clear indication on this treatment and, in this study, the lack of radiological assessment. These have been addressed considering data on Literature reporting the importance of symptoms and clinical evaluation as the key factors to give a correct indication also beyond radiological analysis. |
| Study size | 10 | Explain how the study size was arrived at  **Respected:** Yes  **Comment:** Study size derives from inclusion criteria to have at least 2 years follow-up |
| Quantitative variables | 11 | Explain how quantitative variables were handled in the analyses. If applicable, describe which groupings were chosen and why  **Respected:** Yes  **Comment:** Described in the “Statistical analysis” section |
| Statistical methods | 12 | (*a*) Describe all statistical methods, including those used to control for confounding  **Respected:** Yes  **Comment:** Stated in the “Statistical analysis” section |
|  |  | (*b*) Describe any methods used to examine subgroups and interactions  **Respected:** No  **Comment:** Not necessary as to study design |
|  |  | (*c*) Explain how missing data were addressed  **Respected:** No  **Comment:** Not necessary as to study design |
|  |  | (*d*) If applicable, explain how loss to follow-up was addressed  **Respected:** No  **Comment:** Not applicable |
|  |  | (*e*) Describe any sensitivity analyses  **Respected:** No  **Comment:** Not included as to study design |
| Results | | |
| Participants | 13* | (a) Report numbers of individuals at each stage of study—eg numbers potentially eligible, examined for eligibility, confirmed eligible, included in the study, completing follow-up, and analysed  **Respected:** Yes  **Comment:** Every eligible patient was included in the study and analysed |
|  |  | (b) Give reasons for non-participation at each stage  **Respected:** No  **Comment:** There was only on stage of follow-up for each patient |
|  |  | (c) Consider use of a flow diagram  **Respected:** No  **Comment:** Not needed |
| Descriptive data | 14* | (a) Give characteristics of study participants (eg demographic, clinical, social) and information on exposures and potential confounders  **Respected:** Yes  **Comment:** Included |
|  |  | (b) Indicate number of participants with missing data for each variable of interest  **Respected:** No  **Comment:** Not present |
|  |  | (c) Summarise follow-up time (eg, average and total amount)  **Respected:** Yes  **Comment:** Reported in the Results |
| Outcome data | 15* | Report numbers of outcome events or summary measures over time  **Respected:** Yes  **Comment:** Reported in the Results |
| Main results | 16 | (*a*) Give unadjusted estimates and, if applicable, confounder-adjusted estimates and their precision (eg, 95% confidence interval). Make clear which confounders were adjusted for and why they were included  **Respected:** Yes  **Comment:** 95% confidence interval |
|  |  | (*b*) Report category boundaries when continuous variables were categorized  **Respected:** No  **Comment:** Not present |
|  |  | (*c*) If relevant, consider translating estimates of relative risk into absolute risk for a meaningful time period  **Respected:** No  **Comment:** Not relevant |
| Other analyses | 17 | Report other analyses done—eg analyses of subgroups and interactions, and sensitivity analyses  **Respected:** No  **Comment:** Not necessary as to study design. |
| Discussion | | |
| Key results | 18 | Summarise key results with reference to study objectives  **Respected:** Yes  **Comment:** Stated at the end of the Discussion |
| Limitations | 19 | Discuss limitations of the study, taking into account sources of potential bias or imprecision. Discuss both direction and magnitude of any potential bias  **Respected:** Yes  **Comment:** Included in Discussion |
| Interpretation | 20 | Give a cautious overall interpretation of results considering objectives, limitations, multiplicity of analyses, results from similar studies, and other relevant evidence  **Respected:** Yes  **Comment:** Is the main subject of the Discussion |
| Generalisability | 21 | Discuss the generalisability (external validity) of the study results  **Respected:** Yes  **Comment:** Included in the Discussion, In particular how the findings of this study may affect orthopaedic clinical practice |
| Other information | | |
| Funding | 22 | Give the source of funding and the role of the funders for the present study and, if applicable, for the original study on which the present article is based  **Respected:** No  **Comment:** No source of funding |

*Give information separately for exposed and unexposed groups.

**Note:** An Explanation and Elaboration article discusses each checklist item and gives methodological background and published examples of transparent reporting. The STROBE checklist is best used in conjunction with this article (freely available on the Web sites of PLoS Medicine at http://www.plosmedicine.org/, Annals of Internal Medicine at http://www.annals.org/, and Epidemiology at http://www.epidem.com/). Information on the STROBE Initiative is available at http://www.strobe-statement.org.
